# Gastric cancer and Hedgehog signaling pathway: emerging new paradigms

**DOI:** 10.18632/genesandcancer.168

**Published:** 2018-01

**Authors:** Adamu Ishaku Akyala, Maikel P. Peppelenbosch

**Affiliations:** ^1^ Department of Gastroenterology and Hepatology, Erasmus MC, Erasmus University, Rotterdam, Rotterdam, The Netherlands; ^2^ Department of Microbiology, Faculty of Natural and Applied Sciences Nasarawa State University, Keffi, Nasarawa, Nigeria

**Keywords:** patched, receptors, smoothened receptor, zinc finger protein GLI1, precision medicine

## Abstract

Ever since its initial discovery in Drosophila, hedgehog signaling has been linked to foregut development, The mammalian genome expresses three Hedgehog paralogues, sonic hedgehog (Shh), Indian Hedgehog, and desert hedgehog. In the mucosa of the embryonic and adult foregut, Shh expression is the highest. It has now become clear that hedgehog signaling is of pivotal importance in gastric homeostasis. Aberrant activation of hedgehog signaling is associated with a range of pathological consequences including various cancers. Also in gastric cancer, clinical and preclinical data support a role of Hedgehog signaling in neoplastic transformation, and gastrointestinal cancer development, also through cancer stroma interaction. Technological advance are facilitating monitoring Hedgehog signaling broadening options for the more efficient screening of individuals predisposed to eventually developing gastric cancer and targeting Hedgehog signaling may provide opportunities for prophylactic therapy once atrophic gastritis develops. Nevertheless, convincing evidence that Hedgehog antagonists are of clinically useful in the context of gastric cancer is still conspicuously lacking. Here we analyze review the role of Hedgehog in gastric physiology and the potential usefulness of targeting Hedgehog signaling in gastric cancer.

## INTRODUCTION

Hedgehog proteins are fundamental regulators of embryological development, and tissue homeostasis in adult organisms. Disturbed hedgehog signaling is associated, amongst others, with a range of congenital disabilities, oncological malignancies and immunological defects [1]. Hedgehog proteins intercellular signaling molecules of unusual and fundamental relevance as also illustrated by their substantial conservation across the animal kingdom [2-5]. Initially recognized as a segment polarity gene in Drosophila, now numerous vertebrate paralogues have been found, and in mammals, these include Sonic Hedgehog (Shh), Desert Hedgehog (Dhh), and Indian Hedgehog (Ihh), with Shh being the most comprehensively characterized [5]. Although mainly associated with organogenesis and general and embryological formation of the intestines, in particular, Hedgehog signaling remains active until death and serves to maintain lifelong histostasis in the intestinal tract and also the immune system [6-8]. The pathophysiological importance of Hedgehog signaling is illustrated by the observation that continuous hedgehog signaling is an essential permissive factor in endodermal cancer development [9-11]. With regard to the above, especially the stomach is relevant, where the morphogennot only maintains pit-gland asymmetry, but also fosters the development of gastric cancer, homeostasis, and neoplastic transformation [12-14]. Part of this nefarious functionality is related to the initiation of gastric inflammation due to Helicobacter infection [12]. As stated, although classically associated with gestation, the role of Hedgehog pathway also has important functionality beyond embryogenesis and a potentially vicious one concerning oncological disease. In cancer, both autocrine Hedgehog signaling and paracrine signaling (through the tumor stroma that would thus nurture the tumor cells) of Hedgehog ligands is well-established [15, 16]. Both autocrine and paracrine Hedgehog signaling should be sensitive to pharmacological inhibitors and are thus tested in clinical trials in addition to an intense preclinical research effort [16]. The importance of Hedgehog signaling gastric pathophysiology has led to hopes that pharmacological inhibitors of this signaling may become useful for combating oncological disease in the stomach and this consideration prompted us to review here the detailed molecular mechanism by which Hedgehog influences gastric pathophysiology and to evaluate the evidence that anti-Hedgehog strategies will prove effective in this respect.

The physiological importance of Hedgehog signaling in the physiology of the proximal tract is illustrated by the phenotypes observed in mice with genetic loss of Hedgehog paralogues. Genetic knockout of both Shh and Dhh provoke by malrotation of the gastrointestinal tract, oesophageal atresia, gastric overgrowth and other gross abnormalities [17, 18]. The specific importance of Hedgehog signalling for the stomach in this respect is illustrated by the observation in mice from embryonic day 16 onwards as dichotomy occurs in that the foregut and at the level of antrum and pyloric border region which becomes dramatically more active with respect to Hedgehog signalling as compared to the adjacent duodenal tissues [19], and also is proposed to maintain pit-gland asymmetry in the stomach[7, 20]. Thus the relevance of Hedgehog signaling for gastric physiology seems evident. With regard to pathophysiology, Hedgehog signaling is suggested to be pivotal for gastric cancer progression in both of humans and animals, but a definite etiological role has not yet been shown for this pathway in gastric cancer. To further analyze the precise evidence available in this respect it is essential first to review the molecular details of the molecular signaling involved [21].

### Hedgehog signaling: An overview

**Figure 1:**
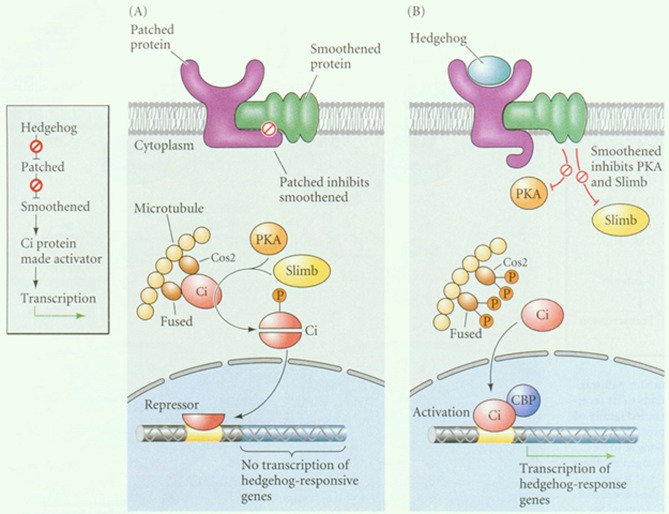
A simplified model of the mammalian Hh pathway In the ‘OFF’ state, Patch inhibits the activity of SMO. Inactive SMO is unable to inhibit Sufu, which promotes processing of the Gli transcription factors in favour of shorter, transcriptional repressor forms (GliR). In the ‘ON’ state, Hh ligands bind to and inhibit Patch, thus releasing SMO activity and in turn blocking Sufu. Gli processing is then shut down, leading to the accumulation of transcriptional activator forms (GlIA).

Hedgehog signaling in general is unusual and complicated, and an immense scientific effort has been necessary to unravel its general principles [16, 22-24]. Signaling is initiated by the different Hedgehog ligands, in casu Shh, Ihh, and Dhh. In the classical Hedgehog signal pathway activation, these different ligands bind a common cognate membrane-bound receptor called Patched that has approximately 1,500 amino acids. The protein transverses the plasma membrane twelve times and thus strongly resembles ABC transporter proteins. In accordance both The N-terminal and C-terminal domains of the protein reside at the cytoplasmic side of membrane, The tertiary conformational of Patched allows Hedgehog ligands to bind via the interaction with two extracellular loops [16, 25]. There are two genes encoding Patched receptors in humans; which are dominated as PTCH1 and PTCH2, and differ slightly concerning their amino acid configuration in the N-terminal region [16, 25]. While both PTCH1 and PTCH2 receptors are associated with numerous human cancers, concerning gastric cancer, especially PTCH1 is the relevant gene product. The function of Patched is to exclude the second receptor, called Smoothed from the primary cilium and retained Smoothened in a vascular compartment/ Binding of Hedgehog to PTCH release this inhibition enabling further downstream signaling [16, 26]. Figure 1 provides a graphical representation.

Following binding of Hedgehog to Patched. Smo translocates to the primary cilium in the cell membrane. The subsequent signaling culminates in altered transcription through Gli transcription factors. The molecular details of Smoothened signaling to Gli is still partly obscure but involves the microtubule transport proteins [16, 27]. The Gli family are members of the Kruppel family of zinc finger transcription factors and a role for three different Gli proteins in Hedgehog signalling has been identified, Gli1, Gli2, and Gli3, each with a distinctive role [16], In the absence of Smoothened activation, GLI1 and GLI2 are transcriptional repressors, but following activation of the pathway these proteins are converted to transcriptional activators [16, 28]. The role Gli3 appears to be mainly as a negative regulator of Hedgehog signaling. It is thus possible to interfere with Hedgehog signaling at different levels, although clinically the use of Smoothened inhibitors has gained the most attention.

### Role of Hedgehog Signalling in Gastric Homeostasis

Ever since its initial detection in Drosophila, Hedgehog has long been associated with foregut development. Of the three mammalian Hedgehogs (3) Shh levels are most highly expressed h, in the mucosa of the embryonic foregut, [29]. Also in other foregut-derived organs such as the lung, Shh expression is prominent, reflecting the embryonal situation [30-33]. Table 1 lists expression patterns in physiology and pathology. Interestingly high Shh expression in the stomach is lost upon the development of intestinal metaplasia (Table.1), suggesting that gastric epithelium-specific effects of the morphogen [12]. Indeed Shh controls gastric epithelial cell maturation and differentiation in the adult stomach[7, 34].

**Table 1:** Small molecules related Hedgehog expression in Gastric Cancer

S/No	Homologs	Normal Intestine	Stomach Metaplasia	Tumor		Techniques	Reference
				Intestinal	Diffuse		
**1**	Shh	Gland Epithelium	Undetectable	elevated	Undetectable	IHC,RT-PCR IHC, RT-PCR RT-PCR,IHC,IMF	[62] [63] [64] [65]
**2**	Ihh	Pit Epithelium	Undetectable	elevated	Undetectable	IHC, RT-PCR PCR,IHC,IMF	[62] [66]
**3**	Dhh	Gland Epithelium	Undetectable	elevated	elevated	IHC, RT-PCR PCR,IHC,IMF	[62] [66]
**4**	Ptch1	Pit Mesenchyme	Detected	elevated	elevated	IHC, RT-PCR RT-PCR, IMF RT-PCR, LacZR	[62] [66] [65]
**5**	SMO	Pit / Gland Mesenchyme	Detected	elevated	elevated	IHC, RT-PCR PCR,IHC,IMF	[62] [66]
**6**	Gli 1	Pit / Gland Mesenchyme	Undetectable	elevated	elevated	IHC, RT-PCR PCR,IHC,IMF	[62] [66]
**7**	Gli 2	Pit Mesenchyme	Undetectable	elevated	elevated	IHC, RT-PCR PCR,IMF	[62] [66]
**8**	Hip	Detected	Not Reported	Undetectable	Not Reported	RT-PCR, IMF	[62, 66]
**9**	BOC	Pit	Undetectable	elevated	elevated	IHC, RT-PCR	[62]

NOTE: NA: not available; Hh: Hedgehog; Gli: glioma-associated oncogene; Ptch: Patched; Smo: Smoothened; Shh: Sonic Hh; IHC = immunohistochemistry, ISH= in situ hybridization, LacZ reporter. RT-PCR real-time PCR.

During progression from the inflamed stomach to gastric cancer, the epithelium goes through defined series of morphological transitions. First, the acid-producing parietal cells are lost and are replaced by mucus-secreting cells that express spasmolytic polypeptide (SP) or trefoil factor 2.7 [35]. Mostly in mice, but also in human subjects, the presence of SP-expressing mucosa (SPEM) defines gland atrophy [36, 37]. Together with atrophy of the parietal cells [33] Shh expression diminishes [38, 39] Although Shh expression diminishes along with the loss of parietal cells [15] the expanding mucous cell compartment or SPEM continues to produce Shh in both human subjects [34, 38] and rodents,[39, 40] but appears to remain as the unprocessed pre-morphogen. Thus functionally expression is lost. Studies suggest that aberrant Hh signaling in cancer functions mainly as either autocrine or paracrine regulator. Especially in stem cell niche Processing of Shh to its active form (19 kilodaltons) in parietal cells becomes compromised in the absence of gastric acid [41, 42] Atrophy of parietal and zymogenic (chief cell) lineages result in hypochlorhydria and reduced serum pepsinogen I (A) levels compared to pepsinogen II (C)[43-49]. These zymogens are proteins encoded by different gene loci that are used clinically to identify pre-neoplastic changes in the stomach [49]. Pepsinogens A and C are converted to the enzymatically active aspartic proteinases, pepsin A and pepsin C, through intramolecular self-cleavage [49, 50]. Pepsinogen A is produced primarily in the mouse corpus by parietal cells, whereas pepsinogen C is mainly produced by both mucous neck and chief cells throughout the stomach h[41]. This result is consistent with the exclusive expression of pepsinogen A in the human corpus and not the antrum, whereas pepsinogen C marks mucous cells of both the antrum and corpus (www.proteinatlas.org). Pepsin A prefers to cleave proteins at hydrophobic and aromatic residues, particularly at phenylalanine (F) when the pH is less than 2. By contrast, pepsin C recognizes a broader consensus site and uses more comprehensive pH spectrum than pepsin A[50, 51]. Explicitly, it's shown using site-directed mutagenesis that pepsin A cleaves the nascent 45-kilodalton Shh polypeptide at residue 200 (SGGCF200|P) to generate the active 19-kilodalton form, whereas pepsin C does not cleave SHH peptide[41]. This may account for the absence of Shh expression in atrophic gastritis.

### Regulation of Gastrin and Gastric Acidity by Shh

Several studies have examined the impact of blocking Hedgehog signaling in vivo, for instance by employing a transgenic mouse that secretes a natural-inhibitor of Hedgehogs called HHIP employing the parietal cell-specific H+, K+-ATPase β subunit promotor [52]. This approach showed that loss of Hedgehog signaling in parietal cells reduces H+, K+-ATPase gene expression and gastric acid secretion [52]. Usually, hypochlorhydria stimulates gastrin gene expression through a decrease in Somatostatin levels [53] Accordingly, increased plasma gastrin occurred in the HHIP transgenic mice, concomitant with reduced somatostatin expression. Both antral G and D cells possess primary cilia, organelles protruding from the plasma membrane, essential for transducing Hedgehog signaling [54, 55]. Therefore, gastric endocrine cells may well be capable of responding directly to Shh. Functionally, this idea is supported by the observation that that transgenic overexpression of GLI2 suppresses gastrin gene expression [56]. Taken together, the production of Shh by parietal cells and the ability of gastric endocrine cells to sense the ligand through primary cilia are consistent with a central role for Hedgehog signaling in the feedback regulation of gastric acidity

### Modes of hedgehog signaling in gastric cancer

Upregulation of Hedgehog signaling pathway is involved in tumor development [57]. De Sauvage and Rubin postulated models for Hedgehog signaling in human cancer development t[57]. The type I cancers are ligand-independent and involve constitutive stimulation of downstream signaling molecules (e,g, loss of Patched), and an example is basal cell carcinoma. Type II are cancers ligand-dependent were both the autocrine, or juxtacrine signaling mechanisms are involved as seen in pancreatic tumors. In type III cancers also ligand-dependency is observed but this type displays paracrine type signaling [57]. Table 1 provides information on these type of tumors in the context of stomach cancer. Remarkably, these models ignore the involvement of non-canonical signaling mechanisms. A number of studies have evaluated the role of cyclin B1 interaction with Patched, in which a Ptch1-cyclin B1 complex is formed at the plasma membrane in a cyclin kinase-1 (Cdk1)-dependent fashion [58, 59]. This results in a reduction in the mitotic index by the separation of cyclin B1/Cdk1 complex from the nuclear machinery resulting in decreased proliferation. Shh binding to patched release the complex and thus fosters cell cycle progression through G2/M phase checkpoint. Obviously, Smoothened inhibitors do not affect this process. Another study documents Hedgehog-independent activation of Patched through the action of proteases and in particular Caspase 3, splitting the C-terminal from Patched [57, 60, 61]. It is likely that such non-canonical signaling contributes to the pro-oncogenic effects of Hedgehog.

### Cross-Links between Hedgehog Signaling, Chronic Inflammation, and Gastric Cancer

As stated, Hedgehog signaling in the stomach plays a significant role in gastric development, homeostasis, and neoplastic transformation [67]. Initially, Shh was somewhat ignored in the context of gastric cancer, despite the evidence that Shh is highly expressed in gastric cancer cell lines [66]. Although increased levels of Shh have been reported in gastric cancers, its specific role in gastric transformation remains elusive but carries significance because of the availability of Hh antagonists. A link exists through the immune system; several studies show that in gastritis the phenotype of infiltrating myeloid cells changes over time to become myeloid-derived suppressor cells (MDSCs) and that this phenotypic switch requires Hedgehog signaling. More specifically, expression of GLI1, which targets Slfn4 (mice) and SLFN12L and SLFN5 (humans), is an early marker for chronic inflammation-associated myeloid cells in their transition towards the MDSC phenotype. As MDSCs are essential for immune-evasion for transformed cells, Hedgehog signaling can thus favor neoplastic development.

### Hedgehog Signaling pathway inhibitors

**Figure 2:**
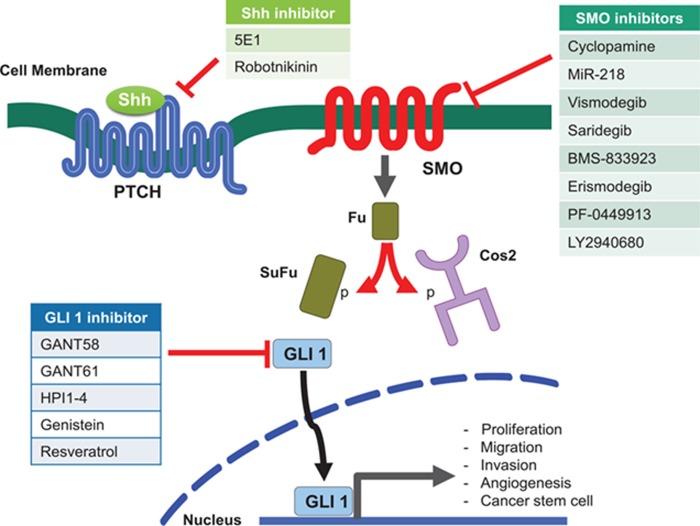
Molecular sites targeted by Hh signaling pathway inhibitors These inhibitors target different components of Hh signaling, including Shh, SMO, and GLI1. These encompass natural compounds, their chemical derivatives, a monoclonal antibody, and chemicals identified from screening libraries.

The Hedgehog signaling pathway is a significant target for cancer therapy. Various molecules that may inhibit the pathway have been evaluated in both preclinical and clinical studies. These inhibitors include: SMO inhibitors, ligand-receptor inhibitors, Gli targeted inhibitors, and these classes of molecules are Illustrated in figure 2.

### SMO Inhibitors

Cyclopamine, originally isolated from the flower veratrum californicum that causes congenital disabilities when eaten by pregnant cattle, was the first compound that was used as a Hedgehog inhibitor and it targets SMO. Consequently, GLIs activation is inhibited. In the clinic, it is side effect-prone and exhibits substantial toxicity [68]. Frustratingly, mice studies involving rhabdomyosarcoma and osteosarcoma models, reveal no significant impact on cancer cells metastasis or growth [69, 70]. From mouse model studies, skin ulcerations and scrubby coat were reported as particularly noticeable skin toxicity; halted even studies initiated to ascertain cyclopamine therapeutic dosing [70]. Adverse effects of cyclopamine in conjunction with other limitations such as acid sensitivity and poor solubility have now halted its clinical development as a potential compound for the treatment of cancer and prompted efforts aimed at. Identifying molecules potentially more suited. This led to the development of the acid stable and water-soluble compound vismodegib (GDC-0449), which was eventually approved by the FDA for the treatment of advanced (Locally), recurrent and metastatic skin cancer. Another semisynthetic novel analogue of cyclopamine (Saridegib (IPI-926) was developed with enhanced potency and metabolically stable[71]. Other inhibitors that impede SMO include LEQ506, PF-0449913, LDE-225 (erismodegib), and BMS-833923. It is important to note that such compounds will not impair Patched-dependent Smoothened-independent non-canonical signaling.

### GLI inhibitors

Also in view of resistance development against vismodegib through SMO mutation efforts have been made to target Gli. Two inhibitory compounds (GANT 58. And GANT 56) were identified through cellular screens aimed at identifying compounds able to inhibit transcription mediated by GLI. Both compounds at the cellular level have been revealed. Understanding the mechanism of action of these compounds at a molecular level is still incomplete. GLI1 posttranslational modification by GANT 61 impedes binding to DNA or changes the conformational structure of the GLI1-DNA complex. In xenograft mice, models of human prostate cancer cells inhibition of cancer cell growth is observed [72]. A study by Hyman et al. also succeeded in identifying four further Hedgehog inhibitors apparently acting downstream of Smoothened: HPI-1, HP-2, and HP-3 are thought to inhibit signaling by targeting a posttranslational modification of GLI or interaction between GLI and a co-factor. HPI-4 was considered to be the only agent that acts by perturbing ciliogenesis, although the mechanism by which HPI-4 disrupts ciliogenesis was not clarified [73]. Again, it is important to note that such compounds will not impair Patched-dependent Smoothened-independent non-canonical signaling

### ligand-receptor interactions disrupting Agents

A monoclonal antibody 5E1 (ch5E1) from murine-human chimeric has been proven to bind Shh and thereby to inhibit Hedgehog signaling[74]. A small molecule (Robotnikinin) was also identified as a Hedgehog signaling inhibitor in a microarray-based screening effort. Robotnikinin binds to Shh thereby inhibiting the activation of Hedgehog signaling [75]. Other new ligand processing blocking agents have been identified in high-throughput screens and appear to have different mechanisms of actions, including interfering with Shh palmitoylation by targeting Hh acyltransferase [76, 77]. These molecules have obvious potential as they also target non-canonical Hedgehog signaling.

### Gastric cancer Treatment using Hh pathway targeted agents

For gastric cancer treatment using Hh signaling targeted agents, only two clinical trials have been performed. A phase II multi-centered, randomized, a prospective clinical trial involving 124 participants was performed to determine the vismodegib efficacy, potency, and safety. For adenocarcinoma patients under FOLFOX chemotherapy, vismodegib an SMO inhibitor was administered in conjunction with this regimen. The study did not meet the primary endpoint with a no significantly-improved progression-free survival between the placebo group and vismodegib group (9.3 months vs. 11.5; p = 0.34), although with a noticeable tendency for prolonged progression-free survival [78]. The lack of a statistically significant result may well stem from this study being underpowered. Lack of good biomarkers that can act as surrogates for progression-free survival can also be considered a confounding factor. CD44 immunopositivity has been established as a biomarker of gastric cancer stem cells. CD44 expression was analyzed in phase II clinical trial samples of gastric tumors and were associated with improved survival. Patients who received chemotherapy alone had poor survival together with high CD44 suggesting a potential role for CD44 as a biomarker in the treatment of patients with Hedgehog signaling targeting agents [79]. Also a BMS-833923 maximum tolerable dose phase 1 clinical trial has been performed. Capecitabine and cisplatin were used in combination with BMS-833923 in drug naïve adenocarcinoma patients. The study was completed in 2013, but the findings have not yet been reported (NCT00909402). Thus a potential role for Hedgehog inhibition in gastric is far from evident and requires more clinical testing.

### Clinical Applications

Gastric cancer has long been seen as one of the most difficult gastrointestinal malignancies to treat. Encouragingly, recent progress with targeted therapies offers hope for patients with advanced gastric cancer and is substantially expanding the therapeutic armamentarium with regard to this infaust disease. As these treatments continue to be developed, we must focus on determination of predictive markers, and preferably co-develop drugs with these markers. The mechanisms underlying primary or acquired resistance to targeted agents also should be clarified to help further drug development[12]. Developing anti-Shh monoclonal antibodies as Shh antagonists is an area to explore where Hedgehog signaling pathway can be blocked at different levels [80]. Gastric cancer is a multigenic disorder influenced by Helicobacter pylori infection and salt intake r. Single nucleotide polymorphism (SNP) and copy number polymorphism (CNP) of genes encoding Hedgehog signaling molecules would be utilized for genetic screening of gastric cancer. Also, cDNA-PCR, microarray, and ELISA detecting aberrant Hedgehog signaling activation would be used for optional therapeutic choice. Genetic testing and precise selection of therapeutic options would contribute to the realization of personalized medicine. Several limitations account for poor treatment outcomes in gastric cancer patients amongst which include tumor heterogeneity. Due to traditional classification of gastric cancer into two categories in casu undifferentiated and differentiated types, obvious biological differences must exist. Additionally, molecular subgroups exist gastric cancer category; these include chromosomal instability tumors, stable genomically tumors, unstable microsatellite tumor and Epstein–Barr virus tumor-positive [81]. It is well possible that stratification for subtype is way forward with regard to Hedgehog inhibition for the treatment of gastric cancer.

## CONCLUSION AND FUTURE DIRECTIONS

While there is good evidence that Smoothened inhibition may be useful for a selection of gastric cancers, we feel that its untargeted application on gastric cancer patients, in general, is likely to prove disappointing. In this sense efforts to select patients characterized by unusually high SMO expression in gastric tumor material with high probability to have cancers that are truly dependent on a functional Hedgehog pathway may likely yield positive results. As both approaches are currently being attempted in clinical trials, it should prove interesting to see whether this notion holds true.
